# RETRACTED: Domínguez-Vergara et al. (2023). Zarit Caregiver Burden Interview: Psychometric Properties in Family Caregivers of People with Intellectual Disabilities. *European Journal of Investigation in Health, Psychology and Education*, *13*(2), 391–402

**DOI:** 10.3390/ejihpe16100167

**Published:** 2026-10-20

**Authors:** Julio Domínguez-Vergara, Henry Santa-Cruz-Espinoza, Gina Chávez-Ventura

**Affiliations:** 1 Research Direction, Universidad Tecnológica del Perú, Lima 15046, Peru; 2 Professional School of Psychology, Universidad Autónoma del Perú, Lima 15842, Peru; 3 Institute for Research in Science and Technology, Universidad César Vallejo, Trujillo 13009, Peru

The journal retracts the article titled “Zarit Caregiver Burden Interview: Psychometric Properties in Family Caregivers of People with Intellectual Disabilities” (Domínguez-Vergara et al., 2023), cited above.

Following publication, concerns were raised with the Editorial Office regarding an overlap between this publication (Domínguez-Vergara et al., 2023) and an earlier thesis (Manrique Alvarez & Torres Carrasco, 2021) authored by another researcher.

In accordance with the journal’s standard procedures, the Editorial Office and Editorial Board conducted an investigation, which confirmed substantial overlap between the two works (Domínguez-Vergara et al., 2023; Manrique Alvarez & Torres Carrasco, 2021) without appropriate citation, attribution, or acknowledgment of the earlier thesis. In addition, the relevant institution confirmed that the ethics approval code reported in the article (Domínguez-Vergara et al., 2023) corresponded to a different research project.

As a result, the Editorial Board has decided to retract this paper as per MDPI’s retraction policy.

This retraction was approved by the Editor-in-Chief of the journal *EJIHPE*.

The corresponding author takes responsibility for the concerns identified. The remaining authors have been informed of this retraction and agreed on the decision.
